# An Electronic Health Intervention for Dutch Women With Stress Urinary Incontinence: Protocol for a Mixed Methods Study

**DOI:** 10.2196/13164

**Published:** 2019-07-11

**Authors:** Lotte Firet, Theodora Alberta Maria Teunissen, Huub van der Vaart, Willem Jan Jozef Assendelft, Kim Josephina Bernadette Notten, Rudolf Bertijn Kool, Antoinette Leonarda Maria Lagro-Janssen

**Affiliations:** 1 Department of Primary and Community Care Radboud Institute for Health Sciences Radboud University Medical Center Nijmegen Netherlands; 2 Department of Gynecology University Medical Center Utrecht Utrecht Netherlands; 3 Department Obstetrics and Gynaecology Radboud University Medical Center Nijmegen Netherlands; 4 IQ Healthcare Radboud Institute for Health Sciences Radboud University Medical Center Nijmegen Netherlands

**Keywords:** eHealth, urinary incontinence, women, mixed methods design, pelvic floor muscle training, study protocol

## Abstract

**Background:**

Stress urinary incontinence (SUI) is a common problem with a great potential influence on quality of life. Although SUI can be treated effectively with pelvic floor muscle training (PFMT), only a minority of women with this complaint seek help. An internet-based electronic health (eHealth) intervention could make care more accessible. The Swedish eHealth intervention *Tät-treatment of Stress Urinary Incontinence* offers PFMT and has shown to be effective in reducing symptoms in women with SUI. This intervention might be helpful for Dutch women too, but its adoption needs to be studied as the Netherlands differs from Sweden in terms of geographical characteristics and health care organization.

**Objective:**

The objective of this protocol is to investigate the barriers and facilitators to the adoption of an eHealth intervention for Dutch women with SUI and the effects of this intervention.

**Methods:**

We are conducting an explanatory sequential mixed methods study among 800 Dutch women with SUI who participate in the translated version of *Tät-treatment of Stress Urinary Incontinence*. This eHealth intervention takes 3 months. A pre-post study is conducted using surveys, which are sent at baseline (T0), 3 weeks after baseline (T1), posttreatment (T2), and 3 months posttreatment (T3). After the intervention, semistructured interviews will be held with 15 to 20 participants. The primary outcomes are barriers and facilitators to using the *Tät-treatment of Stress Urinary Incontinence.* This will also be analyzed among groups that differ in age and severity of incontinence. A thematic content analysis of the qualitative data will be performed. The secondary outcomes are: (1) effect on symptoms of urinary incontinence, (2) effect on quality of life, and (3) factors that are potentially associated with success. Effects will be analyzed by a mixed model analysis. Logistic regression analysis will be used to study what patient-related factors are associated with success.

**Results:**

Enrollment started in July 2018 and will be finished by December 2019. Data analysis will start in March 2020.

**Conclusions:**

An eHealth intervention for Dutch women with SUI is promising because it can make treatment more accessible. The strength of this study is that it explores the possibilities for an internet-based-only treatment for women with SUI by using both quantitative and qualitative research methodologies. The study elaborates on existing results by using a previously tested and effective eHealth program. Insight into the barriers and facilitators to using this program can enhance the implementation of the intervention in the Dutch health care system.

**Trial Registration:**

Netherlands Trial Registry (NTR) NTR6956; https://www.trialregister.nl/trial/6570.

**International Registered Report Identifier (IRRID):**

DERR1-10.2196/13164

## Introduction

Stress urinary incontinence (SUI) is a common problem in women, which has a significant impact on their lives. SUI is defined by the International Continence Society as the complaint of any involuntary urinary leakage on effort or exertion, or sneezing or coughing [1]. Other types of urinary incontinence are urgency urinary incontinence (UUI), which is the complaint of an involuntary leakage accompanied by or immediately preceded by urgency, or mixed urinary incontinence (MUI), which is the combination of both stress and urgency incontinence [1]. SUI is the most prevalent type of urinary incontinence, with prevalence figures ranging from 10% to 39% [2]. Urinary incontinence is associated with a negative impact on quality of life and mental well-being [3], and it affects participation in social activities [4].

Despite the availability of effective treatment options for SUI, only a minority (15% to 38%) of women seek help [5,6]. Pelvic floor muscle training (PFMT) is effective and is recommended as the first-choice therapy for SUI [7,8], which can be provided by a general practitioner (GP) or by a pelvic physiotherapist. Various factors prevent women from help-seeking, such as feeling ashamed, considering urinary incontinence as a consequence of giving birth or of ageing, or lack of knowledge about the treatment options [6,9,10]. Furthermore, GPs encounter difficulties in providing adequate treatment to these women [11,12]. They acknowledge that they experience time restrictions in explaining PFMT and that they lack knowledge and skills for dealing with PFMT [11-13]. Thus, improvement of care for women with SUI is needed.

The delivery of web-based self-help therapy, electronic health (eHealth), is expanding rapidly and has proven to be effective for a wide range of health problems [14]. eHealth appears to be well accepted by women because they prefer the anonymity and flexibility of Web interventions [15]. The feasibility of a Web program as the access point for SUI care seemed to be promising [16]. Various Swedish studies have shown eHealth to be both cost-effective and effective in reducing urinary incontinence symptoms [17-19]. In total, 2 randomized controlled trials showed that symptom severity and incontinence-related quality of life improved significantly after women received an internet-based intervention or mobile phone app intervention with PFMT [17,19]. These treatment effects remained stable after a 1- and 2-year follow-up [20,21], and two-thirds of these women were satisfied with the effect after 2 years [20]. Women appreciated the intervention because they felt that their complaints had been acknowledged [22]. eHealth was not a panacea for all women, however, and a group of women (9% to 22%) sought other treatment after they had participated in the intervention [20,21].

The results from these studies cannot be generalized because a country such as the Netherlands differs from Sweden in geographical characteristics and, hence, in the way its health care provision is organized. Compared with the Netherlands, Sweden has a large number of inhabitants who live in rural areas, which can restrict the access to the health care facilities, such as physiotherapy [23,24]. Although the Swedish Health and Medical Services Act states that health services should be close by and easily accessible to all Swedish citizens, this is challenging in rural areas, even more so since the health care system was marketized in 2010 [24]. This challenge in access to care may have stimulated the uptake of eHealth in Sweden, which is reflected by the country’s long tradition of using telemedicine, one of the first eHealth apps [25]. Due to these differences, therefore, it is questionable whether Dutch women need eHealth for SUI.

Therefore, we perform a mixed methods study with *Tät-treatment of Stress Urinary Incontinence*, an internet-based intervention offering PFMT, for Dutch women with SUI. In this study, we investigate barriers and facilitators to the adoption of an eHealth intervention among Dutch women with SUI who receive the intervention. We also investigate the effects of the intervention on urinary incontinence and quality of life. We expect that this study will provide information that will guide health care providers and policymakers in implementing an eHealth intervention for Dutch women with SUI.

The main objective is to investigate barriers and facilitators to the adoption of an eHealth intervention among Dutch women with SUI. The secondary objectives are to examine the effects of the intervention on symptoms of urinary incontinence and quality of life, and to study factors that are potentially associated with treatment success.

## Methods

### Study Design

We use an explanatory sequential mixed methods design to study the barriers and facilitators to the adoption of an eHealth intervention among participating women and to gain an in-depth understanding of their experiences with the intervention [26]. We are also interested in exploring whether the barriers and facilitators differ between women who vary in age and symptom severity. The quantitative strand is an observational pre and poststudy with women who participated in the Dutch version of *Tät-treatment of Stress Urinary Incontinence* [27]. All eligible women are given the opportunity to participate, and data are collected at baseline, during the intervention (3 weeks after baseline), immediately after the intervention (3 months after baseline), and at follow-up (6 months after baseline). After the eHealth intervention has finished, a qualitative study will be conducted with semistructured interviews to gain more insight into the women’s experiences with the intervention. The Consolidated Criteria for Reporting Qualitative Research will be used to report these qualitative results [28]. The CONSORT-eHealth (Consolidated Standards of Reporting Trials of Electronic and Mobile HEalth Applications and onLine TeleHealth) criteria that are applicable to this study will be applied to report our results [29].

### Setting and Study Population

**Figure 1:**
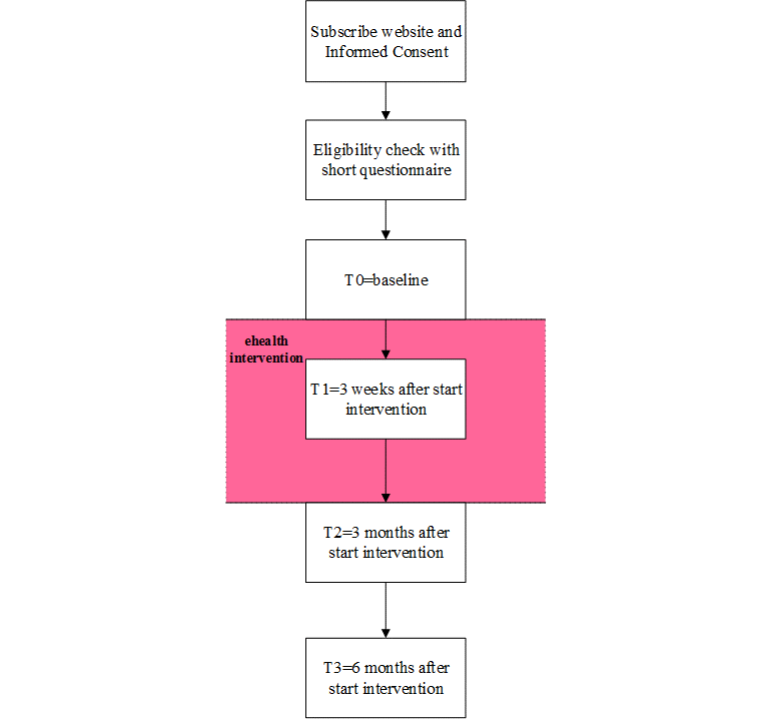
Flowchart of the study. eHealth: electronic health.

Dutch women can subscribe to the intervention on our website (baasoverjeblaas.nl [27]) between July 2018 and December 2019 without needing referral by a health care provider. After providing informed consent (see recruitment and informed consent), they have to fill in a short questionnaire, after which the researcher checks their eligibility (Figure 1).

The following inclusion criteria are applied: women aged >18 years reported having SUI, being capable of understanding Dutch language, and having internet access. Questions to discriminate between different types of urinary incontinence (SUI, UUI, or MUI) were based on the Questionnaire for female Urinary Incontinence Diagnosis, which has proved to be an adequate tool for self-assessment [30]. Women who reported having MUI are included and informed that the intervention is specifically designed for SUI, but that PFMT can also have a positive effect on their UUI component. Eligible women receive the baseline questionnaire, and immediately after completion, they receive a unique token that provides them with access to the intervention.

Women are excluded if one of the following criteria applies: participation in another therapy program or trial for SUI; surgery for urinary incontinence in the last 6 months; PFMT from a pelvic physiotherapist in the last 6 months; pregnancy; vaginal delivery in the last 6 months; neurological disease affecting lower limbs (eg, Parkinson, Multiple Sclerosis, and cerebrovascular incident); and malignancy in lower abdomen currently or in the past 5 years (colon, uterus, cervix, bladder, ovary, or vagina). In case of ineligibility, an email is sent to these women, and, if applicable, we advise them to seek help from their GP or to take a look at a certified self-help website, thuisarts.nl [31]. Excluded women will not be given access to the intervention.

After the intervention, a subset of 15 to 20 women will be asked to participate in semistructured interviews. We will use purposive sampling to study women with variability in age, education level, symptom severity, impact on quality of life, and intervention adherence. We endeavor to interview women immediately after their participation in the intervention to avoid recall bias.

### Conceptual Framework

**Figure 2:**
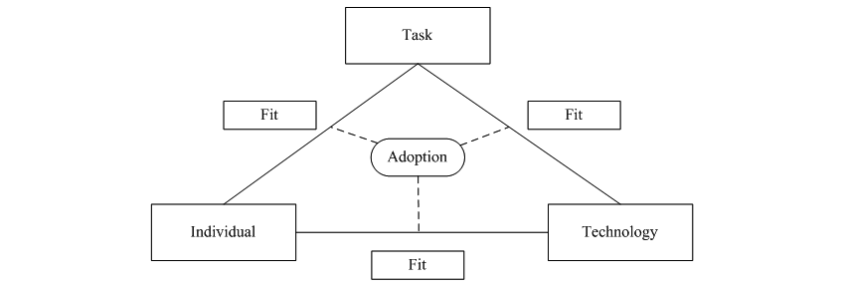
The Fit between Individuals, Task, and Technology framework.

In implementation science, there are multiple outcome variables that can be studied depending on the phase of the implementation, such as acceptability, adoption, feasibility, or sustainability [32]. Various models indicate that an innovation needs to go through multiple stages to make it sustainable [33,34]. The objective of this study is to examine whether the intervention will be adopted by the users. It is known that various factors are determinative for the adoption of new technologies, but the interaction between different factors is also relevant. Ammenwerth et al created the Fit between Individuals, Task, and Technology (FITT) framework, which takes the interaction between different components into account (Figure 2) [35].

According to this framework, the adoption of information technology depends on the fit between attributes of users (eg, motivation and computer anxiety), attributes of technology (eg, usability and performance), and attributes of tasks (eg, complexity). The framework was retrospectively used to study the adoption of a nursing process documentation system and to describe the barriers and facilitators to the 3 attributes in the FITT framework. We will use the same FITT framework in this study to guide our description of the barriers and facilitators to the adoption of *Tät-treatment of Stress Urinary Incontinence*.

On the basis of the known modifiers of PFMT adherence, we hypothesize that the relevant attributes of individuals with regard to this intervention are knowledge, cognitive analysis, planning and attention, and prioritization [36]. Cognitive analysis and planning and attention mean that PFMT adherence depends on the belief of women that the exercise is worth the effort. Furthermore, PFMT requires conscious planning and attention to remember the exercises. The task in our framework will be the participants’ feelings about PFMT and the physical skills they gained during the intervention [36]. On the basis of a behavior change model for internet interventions, finally, we believe that the relevant attributes for technology (the website) are its appearance, behavioral prescriptions, the burden of using the website, training content, and delivery of the message (eg, text and audio) [37].

### Recruitment and Informed Consent

The intervention is an open-access website that enables all women who search for information over Web about urinary incontinence to subscribe to this study. The website provides information about different types of urinary incontinence and about the content of the intervention by means of written text and a video. In addition, we recruit women through advertisements in local papers, posters in waiting rooms of primary care practices or pharmacies, and through other websites displaying a referral link to our website. We explain that the intervention is part of our research project at the Department of Primary and Community Care at the Radboud University Medical Center.

Women who register are requested to fill in their surname (or pseudonym), email address, age, and phone number (optional). Then, they are shown a webpage with information about the study, and they need to tick the *I agree to participate* box at the bottom of that page. This click automatically generates an email with a confirmation link that needs to be clicked to give informed consent. The baseline questionnaire contains 1 item that asks participants for permission to be contacted for an interview after the intervention.

### Intervention

**Figure 3:**
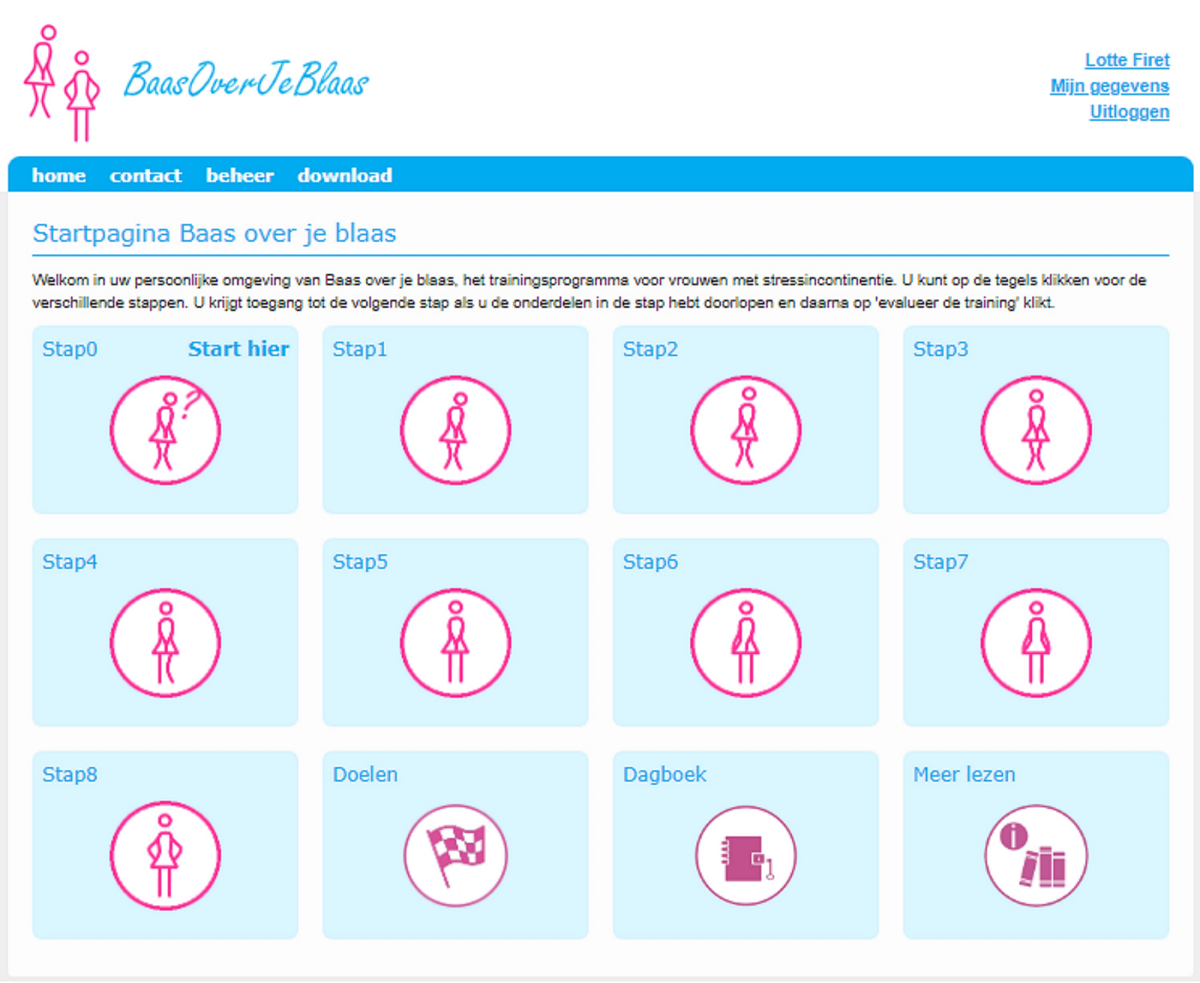
Homepage layout of the Dutch version of *Tät-treatment of Stress Urinary Incontinence*.

The eHealth intervention is based on the Swedish internet-based module named *Tät-treatment of Stress Urinary Incontinence* [38], which was developed at Umeå University in Sweden. The authors (E S, M S, and the eContinence Group) gave their full permission to translate the content, adapt the program to our platform, and conduct an implementation study (noncommercial license by TÄT.nu—eContinence Group on 13 February 2017). The Dutch version of the website was technically constructed by a Web developer and was pilot tested by a group of women who varied in age, education level, and profession. Version 1.0 of our website is currently hosted over Web, and we aim to keep it *frozen* during the entire study period (Figure 3).

**Figure 4:**
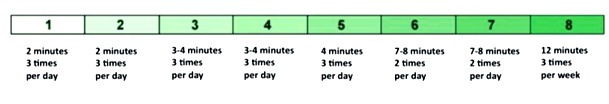
Intensity of pelvic floor muscle training per module in the intervention.

The core content of the website is about PFMT, which is explained by text, audio fragments, and images. Webpages with information and exercises can be downloaded and printed. Next to training, information about urinary incontinence is also provided as well as lifestyle advice to reduce the impact of risk factors for SUI; the negative effect of obesity on SUI, for example, is addressed, and lifestyle advice is provided. In total, 4 different pelvic floor muscle exercises are addressed in 8 escalating modules. Each module contains background information, a training program, and a test exercise that enables women to check whether they gained the correct skills. Depending on the module, participants are recommended to train 2 to 3 times a day for 2 to 12 min, in line with existing guideline recommendations (Figure 4) [7,39].

After completing a module, participants are requested to fill in a training report with 2 questions about the frequency and time they spent on this module. Access to the next module will automatically be provided after the report has been filled in. The intervention will take 3 months, but women can take it at their own pace. Women receive a message 3 months after their first login that their account will be inactivated within a week but that they can download the exercises to continue their training. We decided to inactivate the Web portal to have a cut-off point for the intervention and to achieve a proper measurement of the women’s login activity.

During the intervention, there is no face-to-face contact, but the researcher (a GP in training and researcher) is available for both content-related and technology-related questions through a secured email system. Technology questions are discussed with the Web developer. To stimulate adherence, email reminders are sent if participants do not log-in for 1 week. The content of the reminder is related to the content of the module in which participants are training at that time. A maximum of 2 reminders for each module will be sent, and women are able to unsubscribe themselves. Women who notice no effect of the training or who are unable to contract their pelvic floor muscles are advised to consult their GP for further treatment. This is explained in the intervention as well as in the outro of the questionnaire that is sent 3 weeks after the start of the intervention.

The website is password-protected and allows participants to create their own portal where personal learning goals or training reports can be filled in. Women can create or reset their own password. The eHealth intervention is provided at no cost to participants, and they are not reimbursed for participation.

### Outcomes of Interest

The primary outcome of the study will be the barriers and facilitators to the adoption of *Tät-treatment of Stress Urinary Incontinence*. This outcome will be examined from the perspective of women participating in the eHealth intervention. The secondary outcomes are the effects of the interventions and have been divided into the following 3 items: (1) the effect of an eHealth intervention on symptoms of urinary incontinence, (2) on quality of life, and (3) factors associated with a successful treatment defined by the first secondary outcome.

### Data Collection

We will collect quantitative and qualitative data from women who participated in the intervention (Table 1).

### Questionnaires

Participants are asked to fill in Web-based questionnaires at baseline and at 3 weeks, and 3 and 6 months after baseline (T0, 1, 2, and 3, respectively; Figure 1). They receive a link to the Web questionnaire by email, and they can save their answers and complete the survey at another point in time. Questionnaires can only be completed if all required fields are filled in, and the survey is locked after that, allowing no changes. These data are collected and stored by Castor EDC [40], which is a certified cloud-based Electronic Data Capture platform. Participants who do not complete the questionnaire within 1 week receive a reminder by email.

#### Primary Outcome: Barriers and Facilitators

The barriers and facilitators to the adoption of the eHealth intervention are evaluated by means of questionnaires sent before, during, and after the intervention (T0-T3; Table 1). T0 includes questions about demographic characteristics, medical background, previous help-seeking behavior, and treatment for SUI. Reasons for not seeking help are explored with an open question.

The questionnaire during and after the intervention (T1-T2) contains 5 closed questions about the understandability of the training information (2 times), adherence to the intervention, conditions that would enhance adherence (if applicable), and the possibility to ask questions during the intervention. Some of these questions contain response options that trigger a follow-up open question, for example, to explore reasons for nonadherence or low adherence. T1 and T2 also include 5 closed questions about positive and negative experiences with the intervention (both asked twice) and about suggestions for the intervention’s further improvement.

After the intervention (T2 and T3), we ask women multiple, self-generated questions to evaluate if they sought help since the start of the eHealth intervention or if they intend to seek help from a health care professional for urinary incontinence. The health care professionals who are mentioned in the answer options are the GP, the pelvic physiotherapist, or the specialist (urologist or gynecologist). Reasons for either seeking or not seeking help are explored with open questions that show up depending on the participants’ response to previous questions. We also assess whether participants receive another treatment during or after the eHealth intervention, and, if applicable, what kind of treatment this was. Nonresponders are approached by email first, and, second, by telephone to explore their reasons for not completing the questionnaire. They are also asked if they have any suggestions for improvement.

#### Secondary Outcome: Effect of the Electronic Health Intervention on Symptoms of Urinary Incontinence

At baseline (T0), the situation regarding urinary incontinence is assessed by self-generated questions about symptom duration and about perceived discomfort by a 7-point Likert scale. The effect of the eHealth intervention on incontinence severity is assessed by the International Consultation on Incontinence Questionnaire Short Form (ICIQ-UI SF) at T0, T2, and T3. The ICIQ-UI SF is a validated and a highly recommended 6-item questionnaire to assess the frequency, amount of leakage, and impact on daily life [41]. The total score ranges from 0 to 21, and patients can be divided into 4 categories of severity (overall score: 1-5=slight, 6-12=moderate, 13-18=severe, and 19-21=very severe).

To assess patient-reported improvement, the Patient Global Impression of Improvement (PGI-I) [42] is used. The PGI-I contains one question: “Check the one answer that best describes your urinary incontinence situation, compared with how it was before you began with the study” with 7 response options, ranging from *very much better* to *very much worse*. A successful effect of the eHealth intervention is accomplished if women report that their urinary incontinence is *much* or *very much* improved. This definition is based on the results from a systematic review that reported the definition of success used by studies on both surgical and nonsurgical interventions for SUI [43]. The frequency of using incontinence pads is compared before and after the intervention by one question.

**Table 1:** Data collection. T0: baseline; T1: 3 weeks after baseline (during treatment); T2: 3 months after baseline (posttreatment); T3: 6 months after baseline (posttreatment).

Data categories	Data variables	Quantitative data collection (content of instrument)	Qualitative data collection
Demographic data	Age, education level, marital status, residential area, recruitment method	T0 questionnaire	—^a^
Medical background	Parity, vaginal delivery, gynecological surgery, chronic diseases, defecation problems, symptoms of prolapse, medication use, BMI^b^, smoking, alcohol use, general physical and mental health status	T0, T2, and T3 questionnaires	—
Barriers and facilitators to using eHealth^c^	Positive and negative experiences, understandability, reasons for (non) adherence, support, previous help-seeking behavior, previous treatment received, help-seeking during/after intervention, intent to seek help	T0, T1, T2, and T3 questionnaires	Interview after intervention
Effect of eHealth on urinary incontinence	Baseline information (duration, discomfort) severity, improvement, use of incontinence pads, BMI	T0, T2, and T3 (ICIQ-UI SF^d^, PGI-I^e^) questionnaires	Interview after intervention
Effect of eHealth on quality of life	Urinary incontinence related and general quality of life	T0, T2, and T3 (ICIQ-LUTSqol^f^, Short Form-12) questionnaires	—
Factors associated with success	Age, education level, physical activity, menopausal status, prior surgery for urinary incontinence, (expected) ability to do PFMT^g^, expectation of treatment success, symptom severity, improvement of pelvic floor muscle strength, BMI change, adherence to intervention, adherence to PFMT during intervention, adherence to PFMT after intervention	T0, T1, T2, T3 (ICIQ-UI SF, PGI-I) questionnaires, website data (login data, training reports)	Interview after intervention

^a^Not applicable because data are collected through questionnaire only.

^b^BMI: body mass index.

^c^eHealth: electronic health.

^d^ICIQ-UI SF: International Consultation on Incontinence Questionnaire Short Form.

^e^PGI-I: Patient Global Impression of Improvement.

^f^ICIQ-LUTSqol: ICIQ-Lower Urinary Tract Symptoms Quality of Life.

^g^PFMT: pelvic floor muscle training.

#### Secondary Outcome: Effect on Quality of Life

Quality of life is assessed at T0, T2, and T3 by 2 validated questionnaires: one that is designed specifically for Lower Urinary Tract Symptoms Quality of Life (ICIQ-LUTSqol) and another that is designed for quality of life in general Short Form-12 (SF-12) [44,45]. The ICIQ-LUTSqol contains 19 items about condition-specific issues, such as physical and social limitations relating to incontinence. The total score ranges from 19 to 76, with a higher score implying a greater impact on quality of life. The SF-12 is a shortened version of the SF-36 and aims to assess physical and mental well-being with 12 items. The total score ranges from 0 to 100, with a higher score corresponding to a better quality of life.

#### Secondary Outcome: Factors Associated With Successful Treatment

Several factors will be analyzed for their potential association with intervention effect. The dependent variable is *success*, defined as *very much* or *much* improvement on the PGI-I scale. Characteristics with a potential association are collected at baseline, during, and after the intervention. The content of these characteristics is based on previous studies and includes a wide range of items because the literature is inconsistent on this topic [46-49]. Baseline characteristics that are collected are age, education level, physical activity, menopausal status, previous surgery for urinary incontinence, self-rated ability to do PFMT, expectation of treatment success, and symptom severity (ICIQ-UI SF). Physical activity will be self-assessed using a standard question with 4 levels of activity. The ability to perform PFMT will be assessed on a 10-point Likert scale. The expectation of treatment success will be assessed by using a 5-point Likert scale (1: incontinence definitely stops to 5: no improvement of incontinence), based on the one used by Nyström et al [49].

Characteristics that are collected during and after the intervention include adherence to the intervention, adherence to exercises, symptom severity (ICIQ-UI SF), self-rated improvement of pelvic floor muscle strength, difference in bodyweight compared with baseline, and the frequency of PFMT after intervention. Adherence to the intervention and to the exercises is collected by the website instead of the questionnaire (see website data). Self-rated improvement of pelvic floor muscle strength is assessed during and after the intervention. Participants are asked 3 questions are about their ability to contract the pelvic floor muscles 3 weeks after the start of the intervention (T1). At T2 and T3, participant assess the improvement of their pelvic floor muscle strength on a 5-point Likert scale, with answers ranging from *very much worse* to *very much better* to the question “How is your pelvic floor’s tightening capacity now compared to before the start of the study?” [49]. At T3, 3 months after completing the intervention, participants are asked about the frequency of PFMT with an item that was based on a previous research [46]. Answers to these questions are: *never*, *sporadically, less than once a week*, *regularly, 1-3 times a week*, *regularly, more than 3 times a week*, or *regularly, daily*.

### Website Data

During the intervention, website usage is assessed by collecting website statistics. According to previous research, website usage can be defined by frequency, duration, and activity [50]. Frequency is the number of logins per participant during the 3-month intervention period. Duration is the total time spent on the website, calculated by the time between login and logout, multiplied by login frequency. Activity is the number of Web pages opened within the website. As a logout cannot always be registered accurately, we will not be able to measure duration, and we have chosen, therefore, to approach duration by registering the first and last login data. Frequency is assessed by registering the total number of logins, and activity is assessed by registering the frequency and the type of Web pages that are visited, and the module number that the participants reached.

Adherence to the intervention is defined as the extent to which participants made use of the intervention. We defined 3 groups: nonadherence, intermittent adherence, and continuous adherence, based on previous research [50] and on intervention content. Nonadherence refers to the proportion of participants who never log in after they receive the email with a login token. Intermittent adherence is the proportion of participants who make it up to module 5. Continuous adherence refers to the participants who end up between modules 6 and 8. We set the cut-off point for intermittent and continuous adherence after module 5 as no new pelvic floor exercises are introduced after module 5.

Exercise adherence is measured using the training reports that have to be completed during the intervention to continue to the next module. In each training report, participants need to fill in how many minutes and how often they trained for that particular module. Exercise adherence is defined as the percentage of time spent on PFMT out of expected time spent on PFMT and will be categorized in 3 levels: high (>80%), moderate (20% to 80%), and low (<20%) adherence [48]. The expected time spent on PFMT is based on the prespecified training schedule (Figure 4).

As part of the intervention, participants can fill in their short-term and long-term goals for the training program, and they can make notes in their own diaries, but both are nonobligatory. Both functionalities aim to enhance adherence.

### Semistructured Interviews

After completing the intervention, participants will be asked to participate in a semistructured interview, allowing them to provide feedback in a more narrative form. We undertake to select a subset of 15 to 20 participants with variety in age, education level, urinary incontinence severity, and adherence to the intervention to participate in the interviews. To explore reasons for nonresponse, women who dropped out during the intervention will be interviewed as well. The topics in the interview guide have been divided into the 3 components of the FITT framework [35], a previous qualitative study on eHealth for urinary incontinence [22], and on research group expertise. The following topics will be addressed:

- Individually:
  1. Reason for participation.
  2. Previous experiences with help-seeking, receiving treatment (if applicable).
  3. Expectations of the intervention.
  4. Knowledge of the condition and PFMT.
  5. Cognitive analysis, planning and attention.
  6. Prioritization.
  7. Attitudes toward support during this intervention (advantages/disadvantages of absence of personal contact, attitudes toward email reminders, and suggestions).
  8. Computer skills.
  9. Effects of the intervention (on symptoms and consulting a health care provider).
- Task:
  1. Feelings about PFMT.
  2. Experiences with intervention adherence (including attitudes toward how to enhance adherence, integrating PFMT into daily life).
  3. PFMT complexity (also taking into account previous experiences with PFMT).
  4. Effect on skills gained during the training (ability to contract the pelvic floor muscles).
- Technology:
  1. Appearance.
  2. Behavioral prescriptions.
  3. Burden of using the website.
  4. Content.
  5. Delivery of the message.
  6. Stability of the website and technical problems.
  7. Privacy aspects.

### Analysis

#### Quantitative Data: Questionnaires and Website Data

Descriptive statistics will be used to analyze the characteristics of the participating women, and they will also be described for groups who differ in intervention adherence. The questionnaires include a mixture of open and closed questions that address barriers and facilitators to adopt the eHealth intervention. Responses to open questions will first be analyzed qualitatively, divided into barriers and facilitators, and then be categorized into one of the 3 components of the FITT framework [35].

On the basis of this mapping, different barriers and facilitators will be analyzed for groups that differ in age and urinary incontinence severity with a chi-square test. Treatment effects (T0 vs T2 and T0 vs T3) on the ICIQ-UI SF, ICIQ-LUTSqol, and SF-12 will be analyzed using a mixed model analysis. Missing answers are not likely as Castor EDC does not allow completion of the questionnaire before all required fields have been filled in. Nevertheless, if there are any missing values, they will be replaced with the corresponding answer at baseline and vice versa. If more than 3 answers are missing in a row, the participant will be excluded from further analysis. Descriptive statistics will be used for the analysis on the PGI-I.

Logistic regression analysis will be used to assess the association between different variables and treatment success. Before a definitive model is constructed, the variables will be tested for their unique correlation with the dependent variable, and variables with very-skewed distribution will be excluded or categorized further. Univariate analyses will be performed, and variables with a significance level of *P*<.2 will be included in the multivariate regression model. Variables will then be excluded step by step in order of the highest *P* value until only statistically significant (*P*<.05) variables remain in the multivariate model. The number of variables in the multivariate model will depend on the definite number of participants included in the study. IBM SPSS Statistics 25 software will be used for the analyses.

#### Qualitative Data: Semistructured Interviews, Website Data, and Email

The data that will be collected from the semistructured interviews will be analyzed thematically. With thematic analysis, one strives to identify patterns (themes) that together provide an answer to the research question [51]. Several steps need to be taken to find themes within the data. First, the researchers need to familiarize themselves with the data. They do this by transcribing the audio-recorded semistructured interviews verbatim and by profoundly reading the transcripts. Thereafter, the transcribed interviews will be analyzed using the ATLAS.ti version 8 program. In all, 2 researchers will analyze the interviews independently by applying codes to the transcripts. We will endeavor to compare and discuss the codes after each of the first 3 interviews to check whether the interview guide needs to be adopted. Then, we aim to have the researcher compare codes after 5 interviews. In case of a disagreement, a third researcher will read the transcripts and give his/her opinion. When no new codes emerge, we conclude that data saturation has been reached, which means that no new participants need to be interviewed. Data analysis continues with merging codes into categories. Categories will be discussed in the research team with the aim of constructing themes. During these discussions, the themes will be reviewed and definitions and names for the themes will be constructed.

Document analyses will be conducted with the qualitative data from the emails sent by participants during the intervention and with data from the website (short-and long-term goals for the training and the diary). These documents will not be available from all participants as email contact, diary, and goals are not compulsory.

#### Integration of Quantitative and Qualitative Data

After analyzing the quantitative and the qualitative results, we will combine these results to provide an answer to the primary outcome: barriers and facilitators to the adoption of eHealth for SUI. We will use the FITT framework by describing the *fit* between individual and task, individual and technology, and task and technology [35].

#### Sample Size Calculation

No sample size calculation is needed to provide a reliable answer to our primary outcome. However, a reasonable number of participants is needed to compare groups that differ in barriers and facilitators to the adoption of the eHealth intervention. We decided, therefore, to perform a power calculation based on one of the secondary outcomes: self-rated improvement on symptoms as assessed by the PGI-I. Improvement or success was defined as answering *very much* or *much* to the PGI-I question. Previous studies using the same definition showed that 34% to 56% of participants in PFMT trials improved after the intervention [19,46,47,49]. We used a percentage of 40.9% for the power calculation as this value was derived from the Swedish trial on which our eHealth intervention is based [19]. The power calculation is based on the precision in estimating the percentage of people with very much/much improvement at a certain point in time. To estimate this percentage with a 10% accuracy, the number of 93 women is needed (95% CI 35.9-45.9%).

We expected high drop-out rates as previous research on self-help internet interventions showed rates between 2% and 83% [52,53]. The Swedish eHealth trial *Tät-treatment of Stress Urinary Incontinence* had a 30% drop-out rate. As there is less personal contact during the selection phase in this study, we expected higher drop-out rates, and we took a worst-case scenario into account with a drop-out rate of 80%. To get a number of 100 women completing the questionnaires, therefore, our goal was to include 500 women. After the commencement of this study, we discovered that 40% already dropped out between the moment of registration on the website and the start of the intervention. Therefore, we wrote an amendment to the research ethics committee, who approved our request to include 800 participants.

### Ethical Approval

Ethical approval has been requested and granted (file number 2016-2721) by the research ethics committee of the Radboud University Medical Center, Nijmegen, the Netherlands. This study is conducted in accordance with the Medical Research Involving Human Subjects Act. The committee declared that the risks associated with participation in this trial are negligible according to the Netherlands Federation of University Medical Centers. Handling of personal data will comply with the General Data Protection Regulation (Dutch: Algemene verordening gegevensbescherming).

## Results

Enrollment of participants in the eHealth intervention started in July 2018 and will last until December 2019. Data analysis will start in March 2020.

## Discussion

### Relevance

The mixed methods design in this study allows for a comprehensive and in-depth understanding of the barriers and facilitators to the adoption of an internet-based intervention for women with SUI. The quantitative strand of this study further facilitates an analysis of the effects of the intervention on urinary incontinence symptoms, quality of life, and the factors associated with successful treatment. From previous research, we know that Swedish women were satisfied after using the intervention. Due to geographical and organizational differences with Sweden, however, we do not know whether Dutch women with SUI will also be satisfied with this eHealth intervention. It is important, therefore, to study what factors facilitate or hamper the use of the intervention in the Netherlands. These results can be used to guide health care providers and policymakers in implementing this intervention in the Dutch national health care system. Due to its anonymous and flexible character, eHealth has the potential to improve care for women with urinary incontinence as it lowers the threshold to help-seeking [15]: given that a minority of women with urinary incontinence seek help in regular care [5,6], eHealth might reach those women who would otherwise remain untreated.

### Strengths and Limitations

One of the strengths of this study is that it elaborates on an intervention—proven to be effective—for women with SUI [19]. We translated the content of the program and adapted it to our platform. Although our main objective is not to study the effects of the intervention, we used some of the same validated questionnaires that were used in the Swedish trial to investigate the effect on symptoms and quality of life as a secondary outcome. Another strength is that we used a mixed methods design, which is suitable for describing factors that influence implementation [32]. The use of an existing framework (FITT) further strengthens the study as it helps us include all relevant barriers and facilitators to the adoption of *Tät-treatment of Stress Urinary Incontinence.* This FITT framework will also guide the data analysis.

However, we may not detect all variables as we mostly use open questions in the questionnaire, which do not guide participants to address specific items. We hope to fill this gap with the qualitative strand in this study. Another potential limitation of this study is that we are unable to study the barriers and facilitators among nonresponders or drop-outs, which might lead to selection bias. To mitigate this risk, we have embedded a brief questionnaire at T1 with questions about positive and negative experiences because we expect that more women will respond shortly after registration than at T2 or T3 [53]. Another limitation, finally, could be the absence of a diagnostic procedure by a health care provider. It is known that women can diagnose themselves with the help of questionnaires [30,54], but some women might be mistaken in their diagnosis and take the intervention without effect, causing treatment delay. In this study, we have attempted to decrease this risk using different methods: the researchers check the answers in the selection questionnaire and email those participants whose diagnosis is unclear, women with questions about their diagnosis can contact us by email, and 3 weeks after the commencement of the intervention, we recommend women to consult their GP who are unable to identify and contract their pelvic floor muscles. We believe that it is inevitable that eHealth must be studied without accepting the absence of hands-on diagnostics, but we tried to guide our participants properly by embedding a safety net.

### Conclusions and Implications

In this study protocol, we described the methods for investigating the barriers and facilitators to the adoption of *Tät-treatment of Stress Urinary Incontinence* by patients with SUI. The study expands previous results by using a tested and effective eHealth program. To gain a deeper understanding of the use and uptake of this intervention, we have chosen a mixed methods design. The results of this study are expected to be relevant to policymakers, health care professionals, and patient organizations who play a part in implementing this intervention into the health care system. Insight into the barriers and facilitators to adopting this program can enhance implementation of the intervention in the health care system in the Netherlands.

## Abbreviations

eHealth: electronic health
FITT: Fit between Individuals, Task, and Technology
GP: general practitioner
ICIQ-LUTSqol: Lower Urinary Tract Symptoms Quality of Life
ICIQ-UI SF: International Consultation on Incontinence Questionnaire Short Form
MUI: mixed urinary incontinence
PFMT: pelvic floor muscle training
PGI-I: Patient Global Impression of Improvement
SF-12: Short Form-12
SUI: stress urinary incontinence
UUI: urgency urinary incontinence

## References

[1] Abrams P, Andersson KE, Birder L, Brubaker L, Cardozo L, Chapple C, Cottenden A, Davila W, de Ridder D, Dmochowski R, Drake M, Dubeau C, Fry C, Hanno P, Smith JH, Herschorn S, Hosker G, Kelleher C, Koelbl H, Khoury S, Madoff R, Milsom I, Moore K, Newman D, Nitti V, Norton C, Nygaard I, Payne C, Smith A, Staskin D, Tekgul S, Thuroff J, Tubaro A, Vodusek D, Wein A, Wyndaele JJ, Members of Committees, Fourth International Consultation on Incontinence (2010). Fourth international consultation on incontinence recommendations of the international scientific committee: evaluation and treatment of urinary incontinence, pelvic organ prolapse, and fecal incontinence. Neurourol Urodyn.

[2] Milsom I, Altman D, Lapitan MC, Nelson R, Sillen U, Thom D (2013). International Continence Society.

[3] Abrams P, Smith AP, Cotterill N (2015). The impact of urinary incontinence on health-related quality of life (HRQoL) in a real-world population of women aged 45-60 years: results from a survey in France, Germany, the UK and the USA. BJU Int.

[4] Monz B, Pons ME, Hampel C, Hunskaar S, Quail D, Samsioe G, Sykes D, Wagg A, Papanicolaou S (2005). Patient-reported impact of urinary incontinence--results from treatment seeking women in 14 European countries. Maturitas.

[5] Shaw C, Das Gupta R, Williams KS, Assassa RP, McGrother C (2006). A survey of help-seeking and treatment provision in women with stress urinary incontinence. BJU Int.

[6] Kinchen KS, Burgio K, Diokno AC, Fultz NH, Bump R, Obenchain R (2003). Factors associated with women's decisions to seek treatment for urinary incontinence. J Womens Health (Larchmt).

[7] (2015). National Institute for Health and Care Excellence.

[8] Dumoulin C, Cacciari LP, Hay-Smith EJ (2018). Pelvic floor muscle training versus no treatment, or inactive control treatments, for urinary incontinence in women. Cochrane Database Syst Rev.

[9] Norton JM, Dodson JL, Newman DK, Rogers RG, Fairman AD, Coons HL, Star RA, Bavendam TG (2017). Nonbiologic factors that impact management in women with urinary incontinence: review of the literature and findings from a national institute of diabetes and digestive and kidney diseases workshop. Int Urogynecol J.

[10] Teunissen D, van Weel C, Lagro-Janssen T (2005). Urinary incontinence in older people living in the community: examining help-seeking behaviour. Br J Gen Pract.

[11] Albers-Heitner P, Berghmans B, Nieman F, Lagro-Janssen T, Winkens R (2008). Adherence to professional guidelines for patients with urinary incontinence by general practitioners: a cross-sectional study. J Eval Clin Pract.

[12] Teunissen D, van den Bosch W, van Weel C, Lagro-Janssen T (2006). Urinary incontinence in the elderly: attitudes and experiences of general practitioners. A focus group study. Scand J Prim Health Care.

[13] Shaw C, Atwell C, Wood F, Brittain K, Williams K (2007). A qualitative study of the assessment and treatment of incontinence in primary care. Fam Pract.

[14] Rogers MA, Lemmen K, Kramer R, Mann J, Chopra V (2017). Internet-delivered health interventions that work: systematic review of meta-analyses and evaluation of website availability. J Med Internet Res.

[15] Verhoeks C, Teunissen D, van der Stelt-Steenbergen A, Lagro-Janssen A (2017). Women's expectations and experiences regarding e-health treatment: a systematic review. Health Informatics J.

[16] Barbato KA, Wiebe JW, Cline TW, Hellier SD (2014). Web-based treatment for women with stress urinary incontinence. Urol Nurs.

[17] Asklund I, Nyström E, Sjöström M, Umefjord G, Stenlund H, Samuelsson E (2017). Mobile app for treatment of stress urinary incontinence: a randomized controlled trial. Neurourol Urodyn.

[18] Sjöström M, Umefjord G, Lindholm L, Samuelsson E (2015). Cost-effectiveness of an internet-based treatment program for stress urinary incontinence. Neurourol Urodyn.

[19] Sjöström M, Umefjord G, Stenlund H, Carlbring P, Andersson G, Samuelsson E (2013). Internet-based treatment of stress urinary incontinence: a randomised controlled study with focus on pelvic floor muscle training. BJU Int.

[20] Sjöström M, Umefjord G, Stenlund H, Carlbring P, Andersson G, Samuelsson E (2015). Internet-based treatment of stress urinary incontinence: 1- and 2-year results of a randomized controlled trial with a focus on pelvic floor muscle training. BJU Int.

[21] Hoffman V, Söderström L, Samuelsson E (2017). Self-management of stress urinary incontinence via a mobile app: two-year follow-up of a randomized controlled trial. Acta Obstet Gynecol Scand.

[22] Björk AB, Sjöström M, Johansson EE, Samuelsson E, Umefjord G (2014). Women's experiences of internet-based or postal treatment for stress urinary incontinence. Qual Health Res.

[23] (2018). The World Bank Data.

[24] Kullberg L, Blomqvist P, Winblad U (2018). Market-orienting reforms in rural health care in Sweden: how can equity in access be preserved?. Int J Equity Health.

[25] Olsson S, Jarlman O (2004). A short overview of eHealth in Sweden. Int J Circumpolar Health.

[26] Fetters MD, Curry LA, Creswell JW (2013). Achieving integration in mixed methods designs-principles and practices. Health Serv Res.

[27] Baas over je blaas.

[28] Tong A, Sainsbury P, Craig J (2007). Consolidated criteria for reporting qualitative research (COREQ): a 32-item checklist for interviews and focus groups. Int J Qual Health Care.

[29] Eysenbach G, CONSORT-EHEALTH Group (2011). CONSORT-EHEALTH: improving and standardizing evaluation reports of web-based and mobile health interventions. J Med Internet Res.

[30] Farrell SA, Bent A, Amir-Khalkhali B, Rittenberg D, Zilbert A, Farrell KD, O'Connell C, Fanning C (2013). Women's ability to assess their urinary incontinence type using the QUID as an educational tool. Int Urogynecol J.

[31] Thuisarts.

[32] Peters DH, Adam T, Alonge O, Agyepong IA, Tran N (2013). Implementation research: what it is and how to do it. Br Med J.

[33] Nilsen P (2015). Making sense of implementation theories, models and frameworks. Implement Sci.

[34] Grol R, Wensing M (2004). What drives change? Barriers to and incentives for achieving evidence-based practice. Med J Aust.

[35] Ammenwerth E, Iller C, Mahler C (2006). IT-adoption and the interaction of task, technology and individuals: a fit framework and a case study. BMC Med Inform Decis Mak.

[36] Hay-Smith J, Dean S, Burgio K, McClurg D, Frawley H, Dumoulin C (2015). Pelvic-floor-muscle-training adherence 'modifiers': a review of primary qualitative studies-2011 ICS state-of-the-science seminar research paper III of IV. Neurourol Urodyn.

[37] Ritterband LM, Thorndike FP, Cox DJ, Kovatchev BP, Gonder-Frederick LA (2009). A behavior change model for internet interventions. Ann Behav Med.

[38] (2018). Tät.nu.

[39] Damen-van Beek Z, Teunissen D, Dekker JH, Lagro-Janssen AL, Berghmans LC, Uijen JH, Mientjes GH, Wiersma T (2016). [Practice guideline 'urinary incontinence in women' from the Dutch college of general practitioners]. Ned Tijdschr Geneeskd.

[40] (2016). Castor EDC.

[41] Avery K, Donovan J, Peters TJ, Shaw C, Gotoh M, Abrams P (2004). ICIQ: a brief and robust measure for evaluating the symptoms and impact of urinary incontinence. Neurourol Urodyn.

[42] Yalcin I, Bump RC (2003). Validation of two global impression questionnaires for incontinence. Am J Obstet Gynecol.

[43] Lim R, Liong ML, Leong WS, Yuen KH (2018). Which outcome measures should be used in stress urinary incontinence trials?. BJU Int.

[44] Ware Jr J, Kosinski M, Keller SD (1996). A 12-item short-form health survey: construction of scales and preliminary tests of reliability and validity. Med Care.

[45] Kelleher CJ, Cardozo LD, Khullar V, Salvatore S (1997). A new questionnaire to assess the quality of life of urinary incontinent women. Br J Obstet Gynaecol.

[46] Lindh A, Sjöström M, Stenlund H, Samuelsson E (2016). Non-face-to-face treatment of stress urinary incontinence: predictors of success after 1 year. Int Urogynecol J.

[47] Schaffer J, Nager CW, Xiang F, Borello-France D, Bradley CS, Wu JM, Mueller E, Norton P, Paraiso MF, Zyczynski H, Richter HE (2012). Predictors of success and satisfaction of nonsurgical therapy for stress urinary incontinence. Obstet Gynecol.

[48] Hung HC, Chih SY, Lin HH, Tsauo JY (2012). Exercise adherence to pelvic floor muscle strengthening is not a significant predictor of symptom reduction for women with urinary incontinence. Arch Phys Med Rehabil.

[49] Nyström E, Asklund I, Sjöström M, Stenlund H, Samuelsson E (2018). Treatment of stress urinary incontinence with a mobile app: factors associated with success. Int Urogynecol J.

[50] van den Berg SW, Peters EJ, Kraaijeveld JF, Gielissen MF, Prins JB (2013). Usage of a generic web-based self-management intervention for breast cancer survivors: substudy analysis of the BREATH trial. J Med Internet Res.

[51] Braun V, Clarke V (2006). Using thematic analysis in psychology. Qual Res Psychol.

[52] Eysenbach G (2005). The law of attrition. J Med Internet Res.

[53] Melville KM, Casey LM, Kavanagh DJ (2010). Dropout from internet-based treatment for psychological disorders. Br J Clin Psychol.

[54] Hess R, Huang AJ, Richter HE, Ghetti CC, Sung VW, Barrett-Connor E, Gregory WT, Pinkerton JV, Bradley CS, Kraus SR, Rogers RG, Subak LL, Johnson KC, Arya LA, Schembri M, Brown JS (2013). Long-term efficacy and safety of questionnaire-based initiation of urgency urinary incontinence treatment. Am J Obstet Gynecol.

